# A pilot study investigating human behaviour towards DAVE (Dog Assisted Virtual Environment) and interpretation of non-reactive and aggressive behaviours during a virtual reality exploration task

**DOI:** 10.1371/journal.pone.0274329

**Published:** 2022-09-28

**Authors:** James A. Oxley, Georg Meyer, Iain Cant, Giuseppe M. Bellantuono, Matthew Butcher, Andrew Levers, Carri Westgarth

**Affiliations:** 1 Department of Livestock and One Health, University of Liverpool, Leahurst Campus, Neston, Cheshire, United Kingdom; 2 Institute of Digital Engineering and Autonomous Systems, University of Liverpool, Liverpool, United Kingdom; 3 Virtual Engineering Centre, Daresbury, United Kingdom; Indian Institute of Technology Madras, INDIA

## Abstract

Dog aggression is a public health concern because dog bites often lead to physical and psychological trauma in humans. It is also a welfare concern for dogs. To prevent aggressive behaviours, it is important to understand human behaviour towards dogs and our ability to interpret signs of dog aggression. This poses ethical challenges for humans and dogs. The aim of this study was to introduce, describe and pilot test a virtual reality dog model (DAVE (Dog Assisted Virtual Environment)). The Labrador model has two different modes displaying aggressive and non-reactive non-aggressive behaviours. The aggressive behaviours displayed are based on the current understanding of canine ethology and expert feedback. The objective of the study was to test the recognition of dog behaviour and associated human approach and avoidance behaviour. Sixteen university students were recruited via an online survey to participate in a practical study, and randomly allocated to two experimental conditions, an aggressive followed by a non-reactive virtual reality model (group AN) or vice versa (group NA). Participants were instructed to ‘explore the area’ in each condition, followed by a survey. A Wilcoxon and Mann Whitney U test was used to compare the closest distance to the dog within and between groups respectively. Participants moved overall significantly closer to the non-reactive dog compared to the aggressive dog (p≤0.001; r = 0.8). Descriptions of the aggressive dog given by participants often used motivational or emotional terms. There was little evidence of simulator sickness and presence scores were high indicating sufficient immersion in the virtual environment. Participants appeared to perceive the dog as realistic and behaved and interacted with the dog model in a manner that might be expected during an interaction with a live dog. This study also highlights the promising results for the potential future use of virtual reality in behavioural research (i.e., human-dog interactions), education (i.e. safety around dogs) and psychological treatment (e.g. dog phobia treatment).

## Introduction

Dog bites to humans make up most bite injuries seen in emergency departments in developed countries [1, 2]. Globally, it is estimated that tens of millions of injuries occur each year as a result of dog bites [3]. In England, hospital admission data indicates that the frequency in which people are ‘bitten or struck by a dog’, is increasing, as in 1998 and 2018 dog bite figures per 100,000 were 6.34 and 14.99 respectively [4]. The tripling of adult admissions (from 4.76 in 1998 to 14.99 in 2018) were found to be the reason for this increase as bites to children (≤14 years) were consistently high (mean incidence: 14.44; min: 12.93; max: 15.82) [4]. Two age groups were most frequently admitted due to dog bites including children (1–19) and adults aged between 40 and 49. However, it is important to note that hospital figures are likely to be a vast underreporting of actual dog bite incidence, for example, if medical attention is sought through avenues where dog bite data is not consistently reported (e.g. GP surgery, walk-in centre, accident and emergency department, outpatient data) or an injury is deemed too minor to warrant medical attention (e.g. bruising, superficial wound) [4–7].

To prevent dog bites and reduce the impact for potential victims, owners, dogs and local services (e.g. police, hospitals) we must first understand two contributing factors: 1) the ability of people to recognise and interpret dog behaviour signals; 2) the behaviour of people directly before dog bites occur. Current research generally explores human behaviour that occurs before a dog bite through a variety of methods such as questionnaires [6], interviews [8], police reports [9], online videos [10] and newspaper reports [11]. These methods have limitations, for example, questionnaires, interviews, police reports and newspapers often rely on victims or eyewitness recall of details of the incident (i.e. recall bias). Several studies have investigated human interpretation of dog behaviour in adults and/or children using images and/or videos of dogs [12–14]. However, videos may vary in length, quality (both visual and auditory) and lack control over the context or situation of the aggression, and these studies do not investigate actual behaviour around dogs. To date little research has been conducted on actual human behaviour around a potentially aggressive dog, via direct observations, most likely due to difficulties from an ethical (e.g. dog and human welfare) and context-specific (i.e. lab setting) perspectives [15].

Virtual reality (VR) is a broad term but generally refers to an avenue that enables a user to become psychologically immersed in, and interact (via technology) with, an artificial computer-generated 3D virtual environment [16]. The benefit of this technology is that firstly the environment and its contents (e.g. a dog) can be controlled and modified as needed, and, secondly, the virtual environment, contents and tasks are physically safe [17]. To date, a range of research has used VR to assess challenging virtual scenarios/tasks, such as fire evacuations and pedestrian behaviour and safety during a road crossing, whilst the participant is within a realistic, but risk-free environment [17–19]. VR also allows for users’ behaviour to be monitored and tracked along with physiological responses (e.g., heart rate, skin conductance, etc). VR has been frequently used in the psychological treatment of phobias including animal phobias (e.g., a fear of dogs (cynophobia) [20–22] and spiders [23, 24]). More recently, an AR (Augmented Reality) dog has been developed to assess user awareness, proximity, and locomotion whilst walking a virtual dog [25]. To our knowledge, no research has used VR in the assessment of non-phobic human behaviour for the purposes of understanding human behaviour towards dogs (see scoping review by Oxley *et al*. [26]). Of those dog models that have been developed, largely for dog phobia treatment, little justification or evidence-based research were given regarding the behaviours displayed and various VR equipment and navigation methods were used [26].

Here we present a VR application containing a realistic dog model and environment (known as DAVE (Dog Assisted Virtual Environment) (developed by Virtual Engineering Centre, University of Liverpool, Daresbury, UK)) using a widely cited theoretical framework of dog behaviours, the Canine Ladder of Aggression [27], and assisted with input from three canine behavioural experts (two of which were Board Certified RCVS EVBS (Royal College of Veterinary Surgeons European Specialist in Veterinary Behavioural Medicine), and all three were certified clinical animal behaviourists).

The purpose of this pilot study was to assess the methodology, feasibility, and procedures, of an exploration task within the VR DAVE (Dog Assisted Virtual Environment). We aimed to evaluate the methods for: i) tracking human approach and avoidance towards both a non-reactive and aggressive dog model using the same exploration task, ii) comparing measures of presence (i.e. the experience of being in a virtual world) within a virtual environment between an non-reactive and aggressive model; iii) assessment of participant observation, recognising and understanding of the signs of behaviours based on the Canine Ladder of Aggression.

## Method

### Dog Assisted Virtual Environment (DAVE)

**Fig 1:**
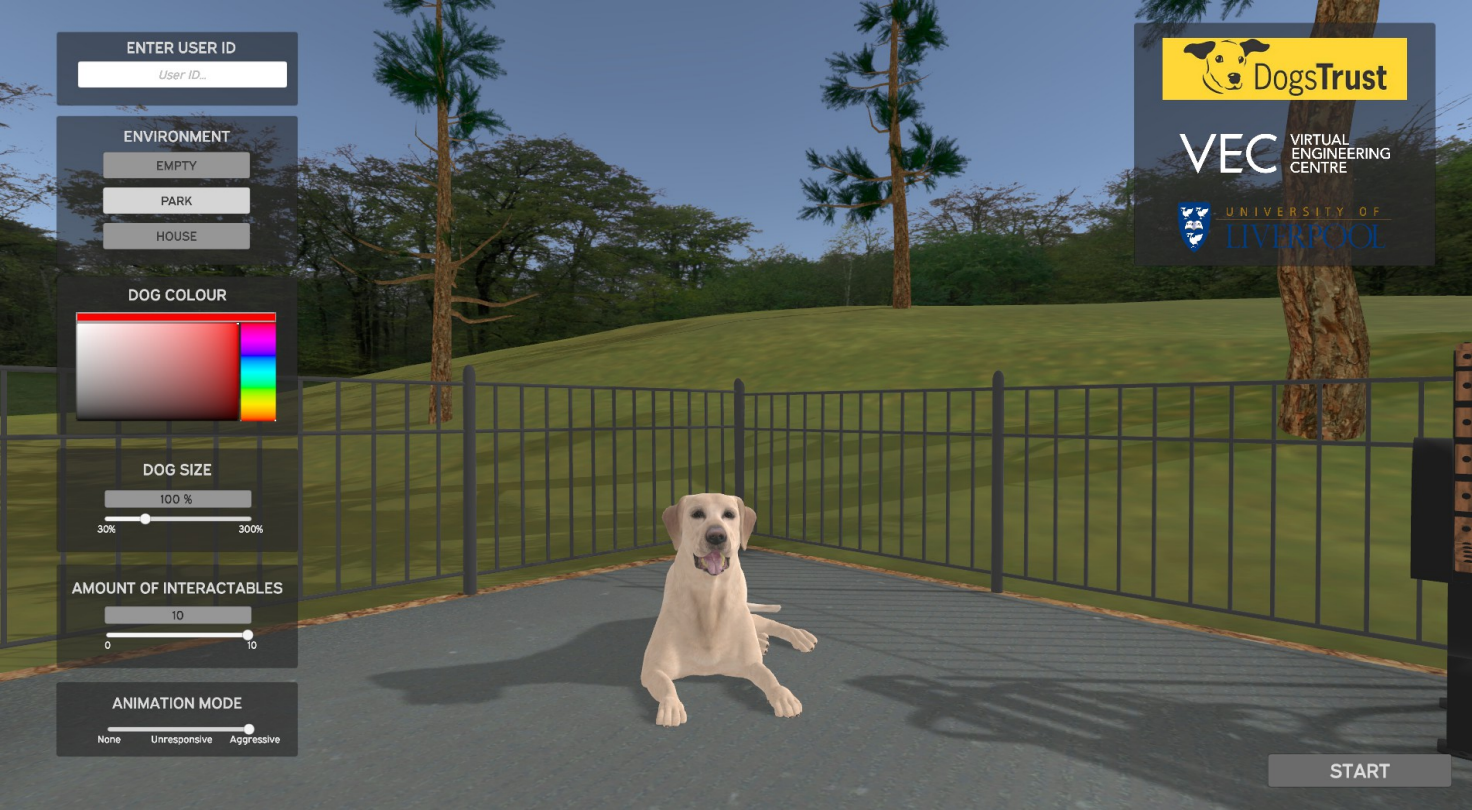
The main menu of the DAVE application.

The dog model (polygons: 29,498; vertices: 29,117) was a Labrador, this breed was chosen due to the common view of the breed being a family dog, the most popular breed in the UK and the multiple coat colours [28, 29]. At the start of the DAVE application, the main menu allows for the customisation of the dog’s coat colour (216 solid colours) and size (30% - 300%). In addition, there are three animation modes (static, non-reactive, and aggressive), three virtual environments (none [blank white space], indoor [living room], outdoor [a park]) and an optional number of collectable items (e.g., mobile phone, remote control) in the environment (0–10) (Fig 1). The setup of the VR environment is based on real-world (or room-scale) walking/movement, regarded as the ‘gold standard’ for immersion in VR [30].

### Apparatus

An HTC VIVE Pro HMD (Head Mounted Display) (resolution: 1440x1600 pixels per eye; refresh rate: 90hz; field of view 110^○^ (VIVE, n.d.)) was used with two HTC VIVE Pro handsets (HTC Corporation, Taiwan). The HMD was connected to MSI GT76 Titan 17.3-inch 4k ultra-HD laptop (Intel Core i9 9900k processor). Both head and hand position coordinates were tracked via SteamVR 2.0 base stations and output through custom Unity software (Unity Technologies, San Francisco), programmed by VEC, Daresbury, UK.

### Virtual dog model scenarios

**Fig 2:**
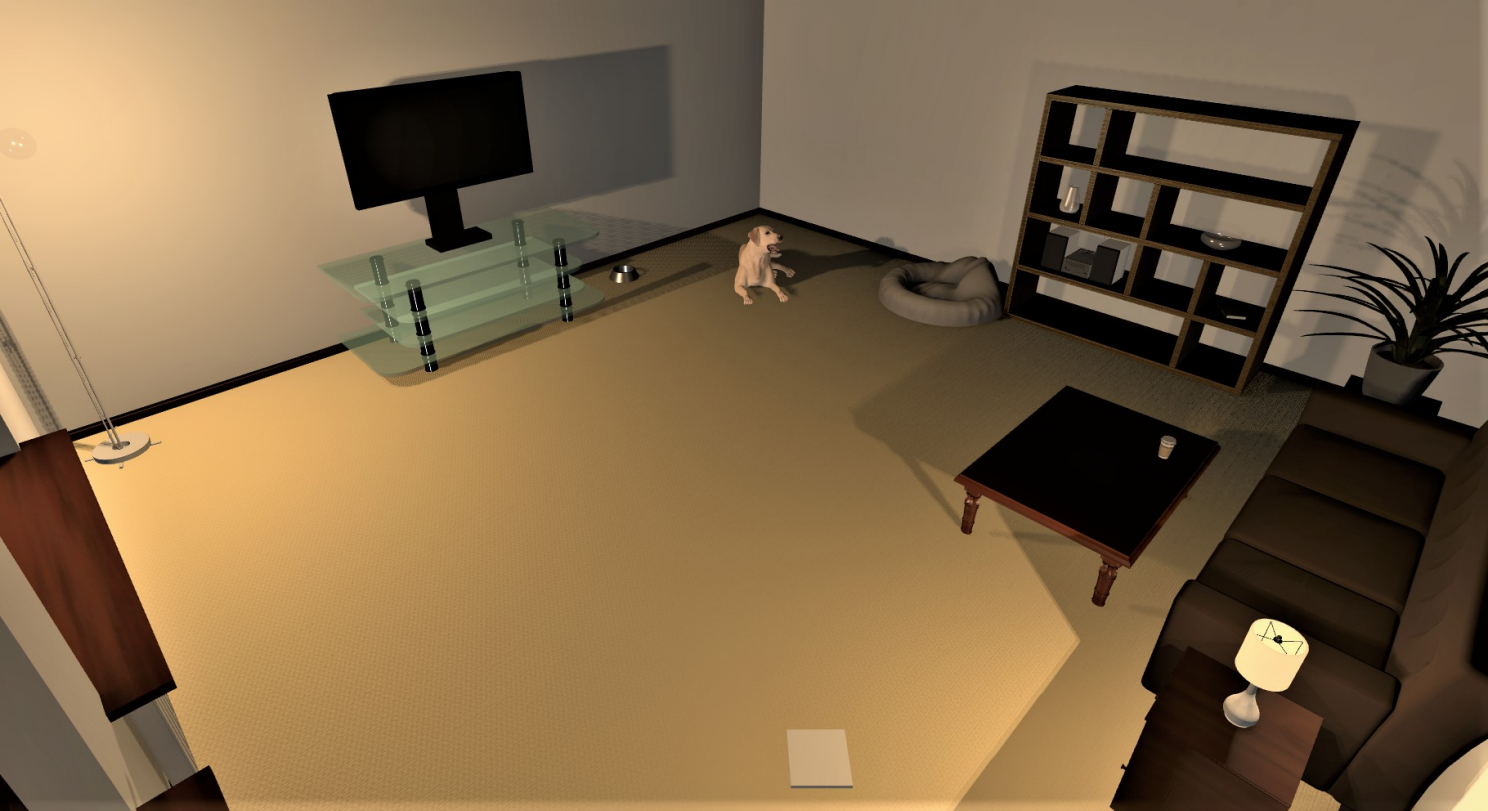
The virtual indoor living room environment.

**Fig 3:**
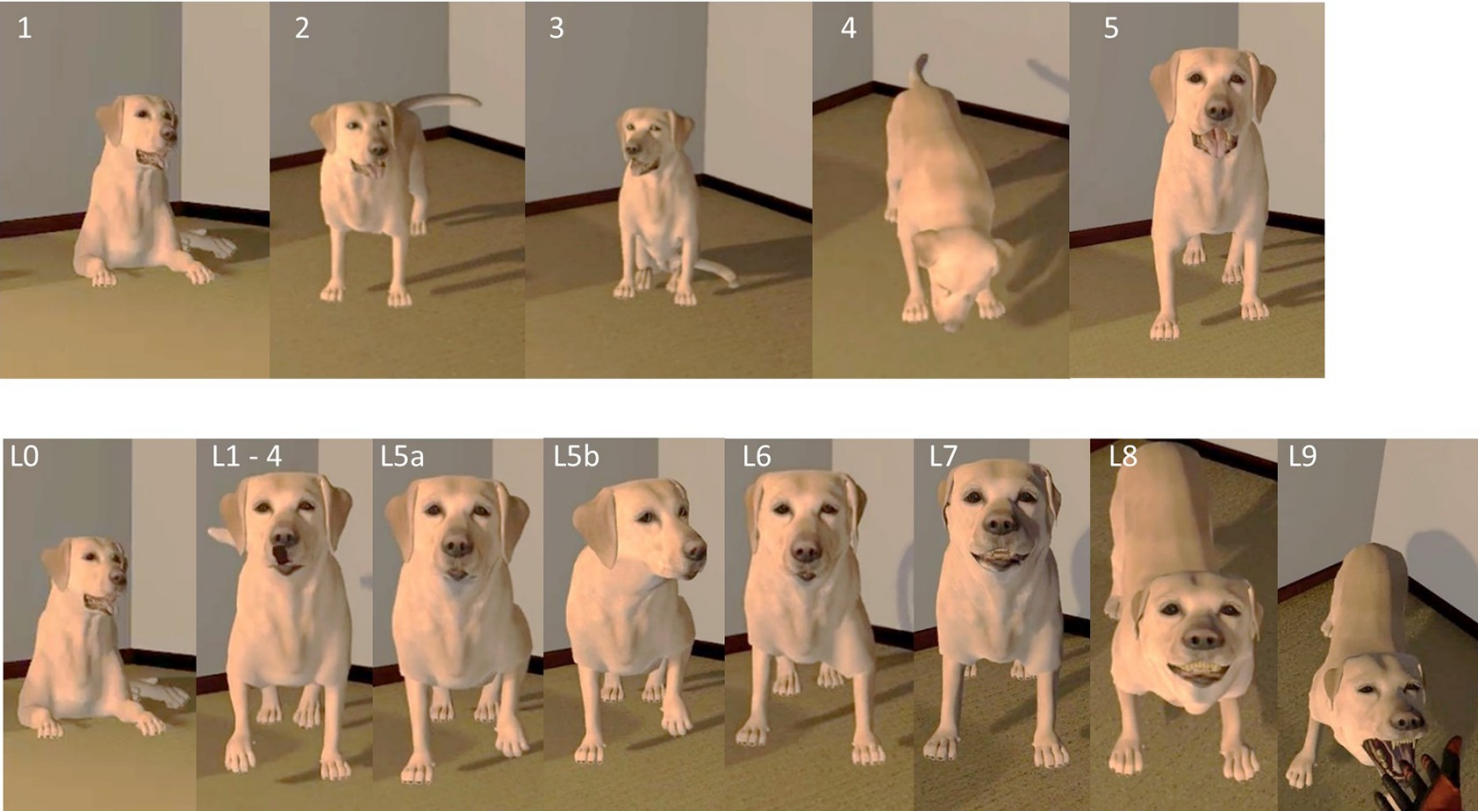
a) Behaviours in the non-reactive scenario; (1) lying down, (2) standing, looking left and right with tail wagging, (3) sitting with mouth open with relaxed tail, (4) Sniffing ground and (5) Step forward with left paw. B) Behaviours in the aggressive scenario at different levels (L); (L0) lying down, (L1-4) lip lick and yawn with increasing frequency, (L5a) paw raise, (L5b) paw raise and head turn. (L6) walking backwards, (L7) crouched with ears back, tail tucked underneath with some teeth showing (L8) crouched, growling, and showing teeth and (L9) Lunge/bite.

**Fig 4:**
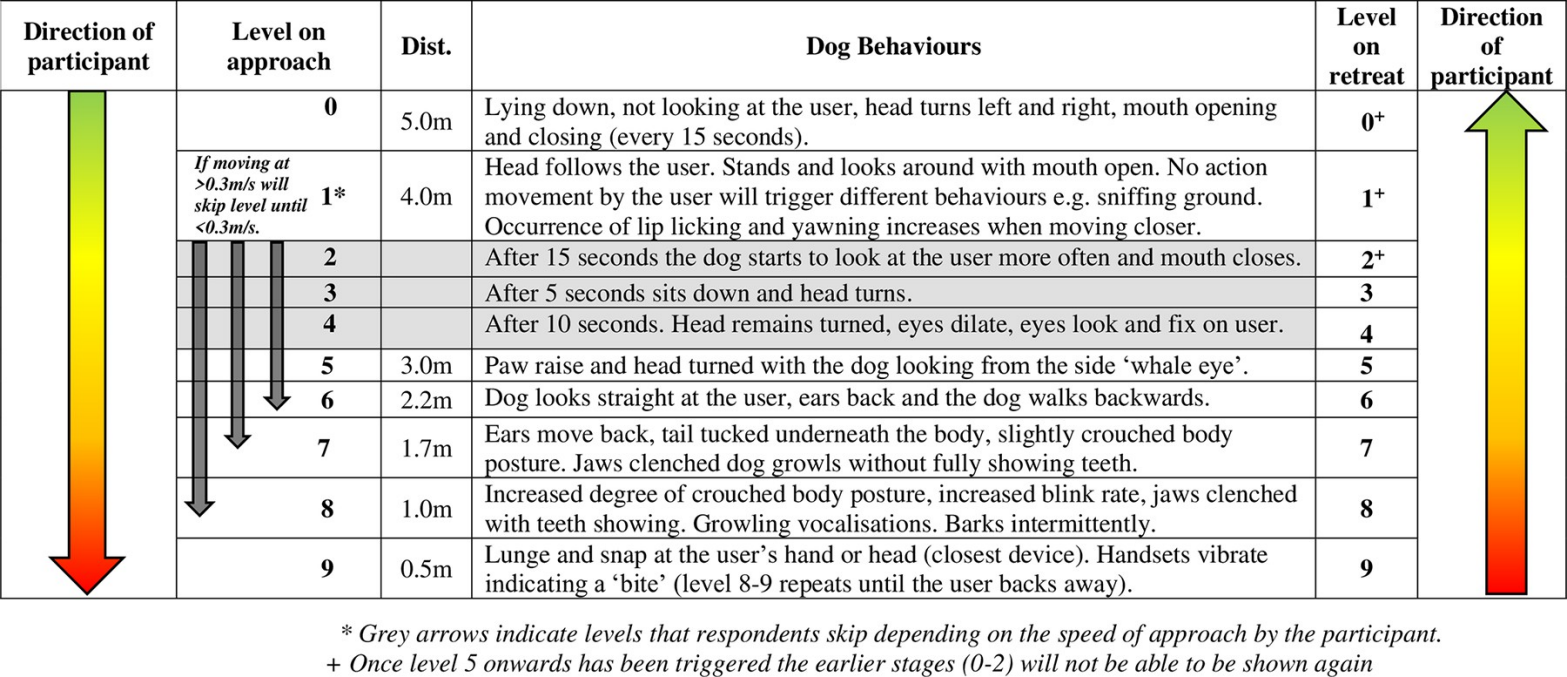
Levels of aggression and behaviours of the dog model (based on the Canine Ladder of Aggression [27]).

For this study, the virtual environment used resembled a domestic indoor living room of a house during the evening (i.e., dark outside, lights on) (Fig 2). The chosen characteristics of the dog model were set as default including size (100%) and colour (yellow). This study included two different dog behavioural scenarios as follows:

- **Non-reactive scenario:** The non-reactive dog model displays a range of neutral behaviours that change over time (irrespective of the participant’s movement or location) as shown in Fig 3A.
- **Aggressive scenario:** The aggressive behaviours are based on the Canine Ladder of Aggression [27] and are dictated by a participant’s distance from the dog and speed of approach (i.e. behaviours are split into 10 levels (0–9) (see Figs 3B and 4) and will display in order (1, 5, 6, 7, 8, 9) if the approach is <0.3m/s per level. If the speed of approach is >0.3 m/s the user will skip levels (e.g., level 1–6, 1–7 or 1–8).

In the aggressive scenario all participants start at level 0 at the beginning of the task. As the user moves forward the location and speed of approach towards the dog will determine the reaction of the dog based on the Canine Ladder of Aggression. Levels of aggression changed automatically based on location of the user within the virtual environment determined by the tracking of the VR headset by the base stations. Once level 5 onwards has been triggered the earlier stages (0–2) would not be shown again. Participants could stop approaching the dog at any point in the task.

### Participant recruitment

**Fig 5:**
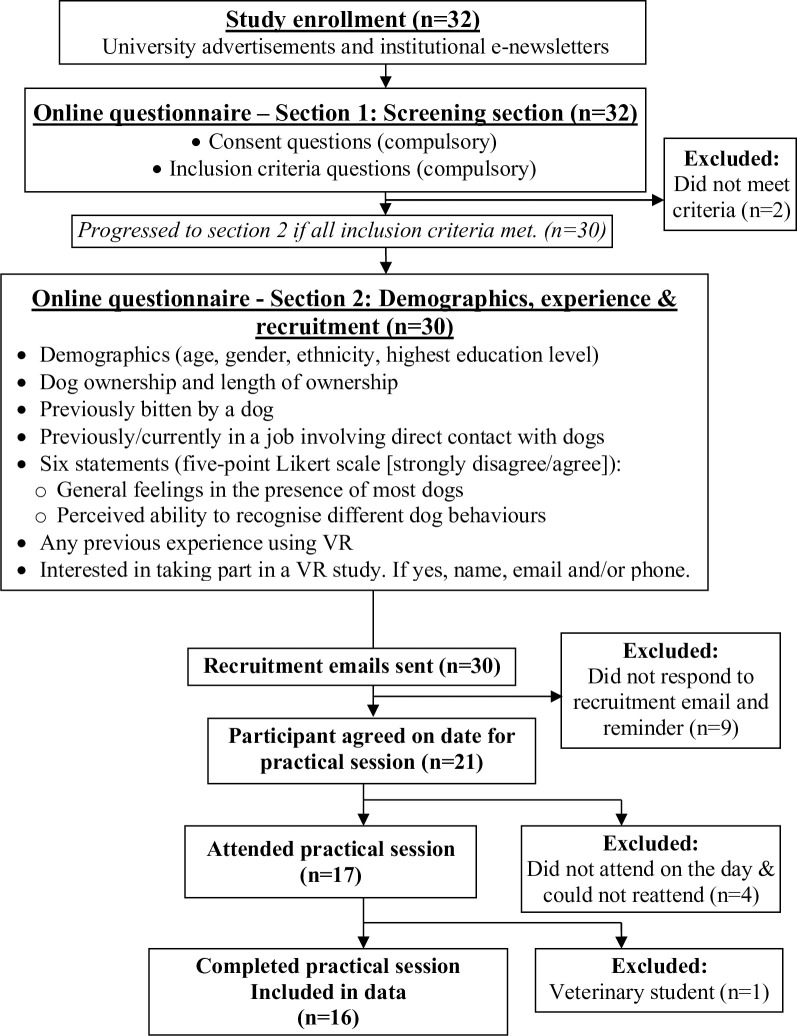
Pilot study screening and recruitment process.

Students were recruited (Fig 5) through an online survey hosted by the survey software JISC, distributed through university departmental newsletters, posters (displayed throughout the university) and the university’s Experiment Participation Recruitment system. Participant inclusion criteria included i) normal or corrected vision (contact lenses/glasses), ii) did not have epilepsy, iii) currently a student at the University of Liverpool, iv) not a veterinary or bio-veterinary science student, v) not scared/fearful of most dogs or have a phobia of dogs and vi) do not feel anxious around most dogs. For respondents who met these criteria information was then collected about demographics, dog-related experience and contact information. Participants were then invited to the study and randomly allocated, using Excel’s ‘RAND’ function, to one of two groups, initially consisting of ten participants per group, each starting with a different scenario first (non-reactive or aggressive) as follows.

- **Group one (hereafter referred to as** **‘NA’****):** Non-reactive scenario first and Aggressive scenario second.
- **Group two (hereafter referred to as** **‘AN’****):** Aggressive scenario first and Non-reactive scenario second.

### Participants

Of the twenty-one students that were recruited, sixteen students, including undergraduate and postgraduate students from the University of Liverpool, aged between 18–35 years (M = 25.19; SD = 5.78 years), took part in the practical session between February and March 2020.

### Task procedure

Upon arrival at the practical task, all participants were asked to read an information sheet explaining the purpose of the study (e.g. “*We are investigating how humans understand dog behaviours and behave around them*”) and data storage, read and sign a consent form and complete a simulator sickness questionnaire (SSQ) [31]. The SSQ was reviewed by the instructor before the start of the task to ensure participants were not already experiencing moderate or severe symptoms (e.g., dizziness).

Participants were introduced to the VR equipment and informed that virtual boundaries of the action area are signalled and were instructed not to go beyond this boundary for safety reasons. The total task area was 6x2m in size. To standardise the starting position participants were asked to stand in a 30x30cm marked area with their heels against the wall. At the start of each task, the participant was directly opposite the dog, 4.6 metres from the dog’s nose. The VR headset was adjusted (i.e. interpupillary distance) to fit each individual participant to ensure a clear image and they were given two VR controllers. In order to assess human behaviour in the presence of the virtual dog models the task for participants was to explore the virtual area. The instruction to “*explore the area*” once the environment appeared was given and the VR application was started by the instructor once the participant was wearing the headset comfortably. Participants had been informed they could stop at any point by either taking their headset off or saying “*stop*”.

Each participant took part in two tasks (see above; NA or AN). A period of five minutes was allocated to each task. After each task participants were asked to complete a post-task survey which included open and closed-ended questions about their perceived meaning of different dog behaviours (S1 Table), a presence [32] and a simulator sickness questionnaire (SSQ) [31]. In total, participants were asked to complete the SSQ on three separate occasions, once before the start of the first task and once after each task.

### Tracking variables

Three-dimensional coordinate data were collected including human head position, left- and right-hand position, dog position (tip of the dog’s nose) and in the aggression scenario, aggression level (0–9). Human head, hand and dog head coordinates (*x*, *y*, *z*) were recorded with a precision of 0.1m at a sampling frequency of 5Hz. Each coordinate position was automatically recorded in Unity software during the live experience and exported in a Microsoft Excel CSV file format.

### Analysis

#### Sample size

As this was the first study using this virtual reality model the required sample size was calculated using a priori power analysis conducted in G*Power software [33]. To detect a large effect size (0.80) [34] between matched pairs assuming normal distribution, a one-tailed t-test with an α-level of 0.05 and statistical power of 0.90, a minimum total sample size of 16 participants was required.

#### Distance travelled and proximity to the dog

Pythagorean theorem was used to calculate the linear distance a participant travelled between two three-dimensional (*x*, *y*, *z*) points. The total linear distance travelled by a participant was calculated as the sum of all absolute linear head movements during the trial. For each participant, the closest proximity a participant’s head and hands came to the dog model was calculated using the Pythagorean theorem to calculate the distance between the participant and dog model (nose) by using their three-dimensional coordinates.

#### Head gaze

Head gaze, (defined as “*head orientation as an approximation of gaze direction*” [35], refers to a ray (or pointer) originating from the centre of the HMD and points in a forward direction which identifies virtual objects the ray intersects (or collides) with, indicating the approximate direction of gaze. The object was automatically recorded in the data output at (every 0.2 seconds (5Hz)) and indicated if the gaze was directed towards the dog or not.

#### Time taken to get to the highest level of aggression

In the aggression scenario, the highest level of aggression (5–9) an individual reached was automatically recorded along with the time it took to reach the level.

#### Statistical analysis

Data management and statistical analysis was conducted in Microsoft Excel and IBM SPSS (Version 27, Armonk, NY: IBM Corp). Boxplots were developed in Origin Pro (OriginLab Corporation, Northampton, MA, USA). Open ended questions were coded and categorised using NVivo 12 (QSR International Pty Ltd).

A Wilcoxon signed rank test was used to compare total distance travelled, the closest distance to the dog, time spent gazing at the dog and total and subscale presence scores, between the aggressive and non-reactive scenario for both the combined data (AN & NA) and individual groups (AN, NA). The size effect was calculated for the closest distance a participant got to the dog (r = z / √ of N) [36]. A Mann Witney U test was used to compare each scenario separately (aggressive and non-reactive) between individual groups for the total distance travelled and time spent gazing at the dog.

To test for associations between the highest level of aggression reached (6–9) and demographic factors a Spearman Rank Test (age) and a Fisher’s Exact Test (gender, dog ownership, previous VR experience) was used.

The presence questionnaire was scored based on the published scales [32]. The total presence score was compared for both combined data and between scenario within each group (NA, AN) using a Wilcoxon signed rank test. The SSQ was scored based on the published scale [31] and completed three times per participant. A Friedmann test was conducted to determine if there was a difference across the three time points (pre-task, task 1 and task 2). A p-value of <0.05 was considered significant.

#### Ethical approval

The study was approved by the University of Liverpool Health and Life Sciences Research Ethics Committee (ref.: 5929). All participants who took part consented to taking part and agreed to the use of the data in the form of a scientific journal article.

## Results

Of the sixteen participants that took part, most were female (12/16) and educated to first degree level or higher (9/16), higher diploma (2/16) or to A/AS level (AS level) (5/16) (Table 1).

**Table 1:** Participant demographics and dog related questions.

Variable	n	%
**Gender**		
Male	4	25.0
Female	12	75.0
**Ethnicity**		
White	14	87.5
Asian/Asian British	2	12.5
**Highest level of education** *		
A/AS level	5	31.3
Diploma in higher education	2	12.5
First degree level qualification	3	18.8
University higher degree (e.g., MSc)	6	37.5
**Current or previous dog ownership**		
Yes, previously but not currently	8	50.0
Yes, currently	2	12.5
No	6	37.5
**Length of dog ownership (n = 10)**		
≥ 9 years	8	80.0
≥ 7 years but < 9 years	2	20.0
**Previously bitten by a dog** *		
Yes	3	18.8
No	13	81.3
**Job involving contact with dogs**		
Yes	2	12.5
No	14	87.5

*Total percentage is 100.1% due to rounding to one DP.

### Dog ownership and related experience

Over half (62.5%; 10/16) of participants either had previously or currently owned dog(s), for seven years or longer. Only two worked in a job with direct contact with dogs and three respondents had previously been bitten by a dog. The majority (>80%) indicated that they were comfortable (i.e., enjoyed (87.5%; 14/16), felt relaxed (81.3%;13/16) and were not cautious (81.3%; 13/16)) in the presence of most dogs and more than 80% felt that they could recognise when a dog is showing aggressive (93.8% 15/16), fearful (81.3%; 13/16) or relaxed (93.8%; 15/16) behaviours (S2 Table).

### Closest proximity to the dog

**Fig 6:**
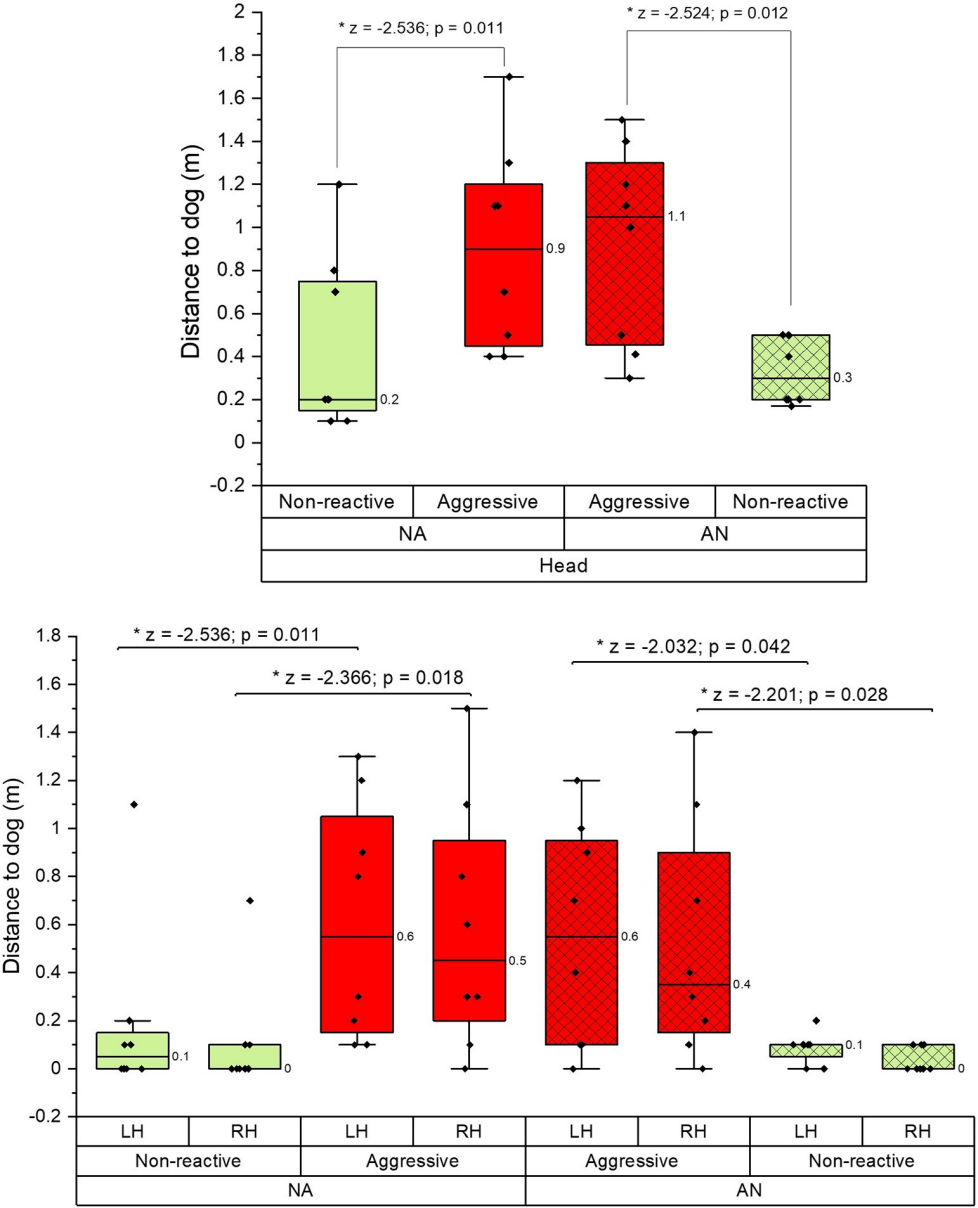
**a)** A boxplot of the closest head position in the non-reactive and aggressive scenarios where the aggressive scenario was first (AN) or the non-reactive scenario was first (NA). **b)** A boxplot comparison showing the closest distance the left- and right-hand got to the dog in non-reactive and aggressive scenarios, where the aggressive scenario was undertaken first (AN) or non-reactive first (NA).

Participants’ head and hand positions moved significantly closer to the dog in the non-reactive scenario than in the aggressive scenario, when all data was combined (NA & AN) (head, left hand, right hand (p≤0.001 and r = 0.8 in all cases)) and separately when comparing within NA and AN groups (p<0.05 in all cases (Fig 6A and 6B)).

### Distance travelled—Head and hand tracking

**Fig 7:**
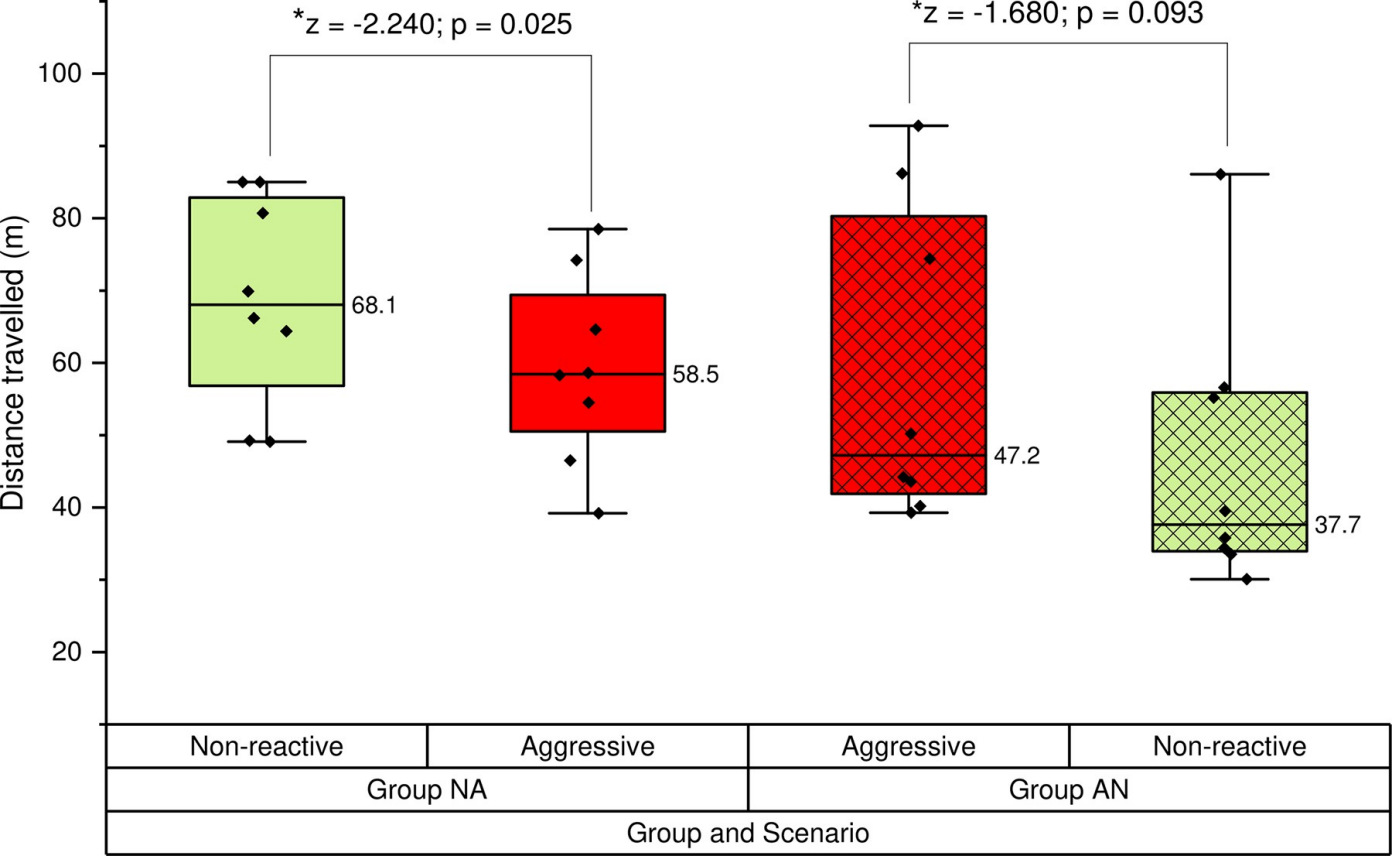
A boxplot displaying the median total distance travelled in each scenario (non-reactive and aggressive) for group NA and AN.

In the combined dataset (both NA and AN) there was no difference in the median total distance travelled between non-reactive (55.9m) and aggressive scenarios (56.4m) (z = -2.59, p = 0.796). The total distance travelled was shorter in the aggressive scenario compared to the non-reactive scenario in group NA (p = 0.025) but not group AN (p = 0.093) indicating an order effect (Fig 7).

There was a significant difference between median total distance travelled during the non-reactive scenario in group NA (68.1m; range: 49.1–85.0m) compared to group AN (37.7m (range: 30.1–86.1m) (Mann Whitney U = 12.00; p = 0.038). There was no significant difference between the median total distance travelled in the aggressive scenario between users in group NA (58.5m; range: 39.2–78.5m) and AN (47.2m; range: 39.3–92.8m) (Mann Whitney U = 28.00; p = 0.721).

### Levels of aggression

In the aggressive scenario, one participant reached level 6, seven reached level 7, five reached level 8 and three reached level 9 (a bite). Participants who moved more slowly towards the aggressive dog also tended to stop at a lower aggression level i.e. at a greater distance from the dog (r = -0.532, n = 16, p = 0.034) (S1 Fig). Most participants (14/16) in both groups spent over 70% of their time between levels 5–9 (S2 Fig). On the first approach, six participants moved from level 1 immediately to 5, five from levels 1 to 6, four from levels 1 to 7 and one participant straight to level 8. Once participants reached level ≥5 the dog would not perform levels 0–2 thereafter (Fig 4).

The was no evidence of association between level reached and age (p = 0.557), gender (p = 0.827), if a person currently/previously owned a dog or not (p = 0.869), if they were previously bitten or not (p = 0.500) or if a person had previous experience of VR or not (p = 0.191). All three participants who reached level 9 (dog lunges and handsets vibrate indicating ‘a bite’) did not currently own a dog and only one had previously been bitten. All three individuals that reached level 9 agreed with the statement that they could recognise aggressive dog behaviours. However, regarding the statement about whether they could recognise a dog showing scared or fearful behaviour one respondent stated ‘disagree’ another stated, ‘neither agree/disagree’ and one stated ‘strongly agree’.

### Head gaze

Individuals that got closer to the dog spent more time gazing at the dog, in both the non-reactive (rho = -0.945; p<0.001) and the aggressive (rho = -0.831; P = 0.011) scenario in group NA only. There was no evidence of significant difference in the time spent gazing in the direction of the dog model between the aggressive and non-reactive scenarios in the combined dataset (NA & AN) (p = 0.121) or individual groups (NA (p = 0.779); AN (p = 0.093)). The median time spent gazing in the direction of the dog model in the non-reactive scenario was lower in group NA than AN (133.5s and 218.4s respectively, Mann-Whitney U = 11.00; p = 0.027), but there was no evidence of a difference in the aggressive scenario (144.0s and 171.7s in groups NA and AN respectively (p = 0.753)).

### Behaviour recognition and interpretation

When participants were asked if they noticed anything about the behaviour of the dog, they most often referred to the movement of the dog’s body as a whole or part of the body (Table 2). Participants frequently used adjectives to describe the emotion or motivation of behaviours of the dog rather than describing actual behavioural signals. In the non-reactive scenario, five noted the tail compared to one in the aggressive scenario. It was also evident that answers differed between the initial open-ended question asking about what a participant noticed about the behaviour of the dog and close ended questions about if they saw a behaviour. For example, in the open-ended question “*Did you notice anything about the behaviour(s) of the dog*”, no participants referred to the early signs of the Canine Ladder of Aggression such as lip licking and only two stated the paw raise. However, when asked about specific behaviours, lip licking (56.3%; 9/16) and head turning (56.3%; 9/16)) were reported albeit not commonly. The most frequently behaviours reportedly seen in the aggressive scenario were raising a paw (100% (16/16)), backing away (93.8% (15/16)), and showing teeth (93.8% (15/16)). All respondents did move through level 1 where the lip lick occurs, however, users admittedly did not spend long there (median 6.5s and 7.7s in group NA and AN respectively). Furthermore, due to the speed of approach, all participants moved from level 1 to ≥5 and after this time level 0–2 was not shown again even if they moved away from the dog, as level 5 had been reached. There was no evidence of a significant difference between time spent in level 1 and if a participant stated they saw the lip lick or not (p = 0.81). Only one person stated they did not see the dog showing its teeth (level 7 onwards), due to this participant being the only person that did not go closer than level 6 and thus did not reach this behaviour. Interpretation of what these aggressive behaviours meant varied but generally agreed that it was some form of negative emotional state such as scared, threatened, or anxious (S3 and S4 Tables).

**Table 2:** Categorised open-ended responses to the question “*Did you notice anything about the behaviour(s) of the dog*” for both non-reactive and aggressive exploration scenario. Multiple descriptions were coded separately (e.g., cowered and bared teeth were coded in two separate categories) per respondent.

Non-reactive scenario–Categories	Count	Aggressive scenario—Categories	Count
**Body**	**12**	**Body**	**17**
**Full body/body movement**	**5**	**Full body/body movement**	**10**
Moving from standing to sitting	3	Moved back/backed away/retreated	7
Moving forward	1	Crouched down, cowered	2
Slow movement without hesitation	1	Sitting	1
**Head**	**2**	**Head**	**4**
Yawning	1	Bared teeth	1
Sniffing	1	Biting, snapping	2
**Tail (wagging)**	**5**	Ears	1
** **		**Paws**	**2**
**General behaviour/emotion**	**9**	Paw raise	2
Alert	1	**Tail (e.g. lowered, between legs)**	**1**
Anxious	1		
Distressed, uncomfortable	1	**General behaviour, emotion, description**	**15**
Friendly	1	Aggressive	3
Placid, bored	1	Agitated	1
Relaxed, calm, happy	3	Did not like people	1
Shy	1	Docile at a distance /initially friendly	2
		Nervous/Scared/Unsure	5
**Vocalisations**	**5**	Threatened	1
Pining, yawning, whimper	2	Uncomfortable	1
Groaning	1	Unfriendly	1
Panting	1		
"No grunting sound"	1	**Vocalisations**	**12**
		Barked	3
**Direction of gaze**	**4**	Growled	8
Looking around	3	Panting	1
Looking at me	1		
		**Location of the dog (e.g. In the corner)**	**1**
**Willing to be petted**	1		
**Distance to the participant**	1		

Regarding the three individuals that were ‘bitten’ (level 9), all 3 individuals stated seeing the dog showing its teeth and that it indicated a threat or warning (“aggression, defensive”, “warning sign, indication to keep away”, “aggressive warning sign”). Two reported noticing the earlier lip lick in the closed ended question, however only one provided an answer as to what it meant “not sure, maybe wanted to wet its mouth”.

All 16 participants stated they heard some form of dog vocalisation during the aggressive exploration scenario. Most reported vocalisations including growling or barking (Table 3).

**Table 3:** Vocalisations reported by participants in an open-ended question post aggression scenario (multiple responses per person).

Vocalisation	Example response	n	%
Growling	*"Growling—a warning sound the dog isn’t happy"*	14	87.5
Barking	*"…Barking when I got near to intimidate me into leaving…"*	12	75.0
Snarling	*"Snarling… - aggressive*, *angry"*	1	6.3
Yawning	*"Yawning and noises with it*. *I’d interpret as signs of anxiety"*	1	6.3
"squeaking"	*" Squeaking—uncomfortable experience and anxious"*	1	6.3
"Grunting"	*"Regular grunting with occasional breaks*. *The grunting seems to be his normal breathing sounds which became quicker as I approached the dog…*.*"*	1	6.3
“Whining”	*"…Whining when I walked to show he was upset"*	1	6.3

### Simulator sickness questionnaire

Findings from the SSQ were low; with seven out of sixteen participants (43.8) scoring 0 (no symptoms) across all three surveys. Of those that did score above 0 (56.2%; 9/16), no rating went above 1, indicating a ‘slight’ symptom. Of these, only 4 participants increased the total score between the pre-test and post-test indicating that there was no increase in total score between pre-test and post-tests for five participants. Mean total scores over the three surveys were all under 10 indicating ‘minimal symptoms’ (S5 Table). The following seven symptoms scored zero over all three surveys for all participants: headache, increased salivation, “fullness of head”, dizziness (closed eyes), vertigo, “stomach awareness” and burping.

### Presence questionnaire

**Fig 8:**
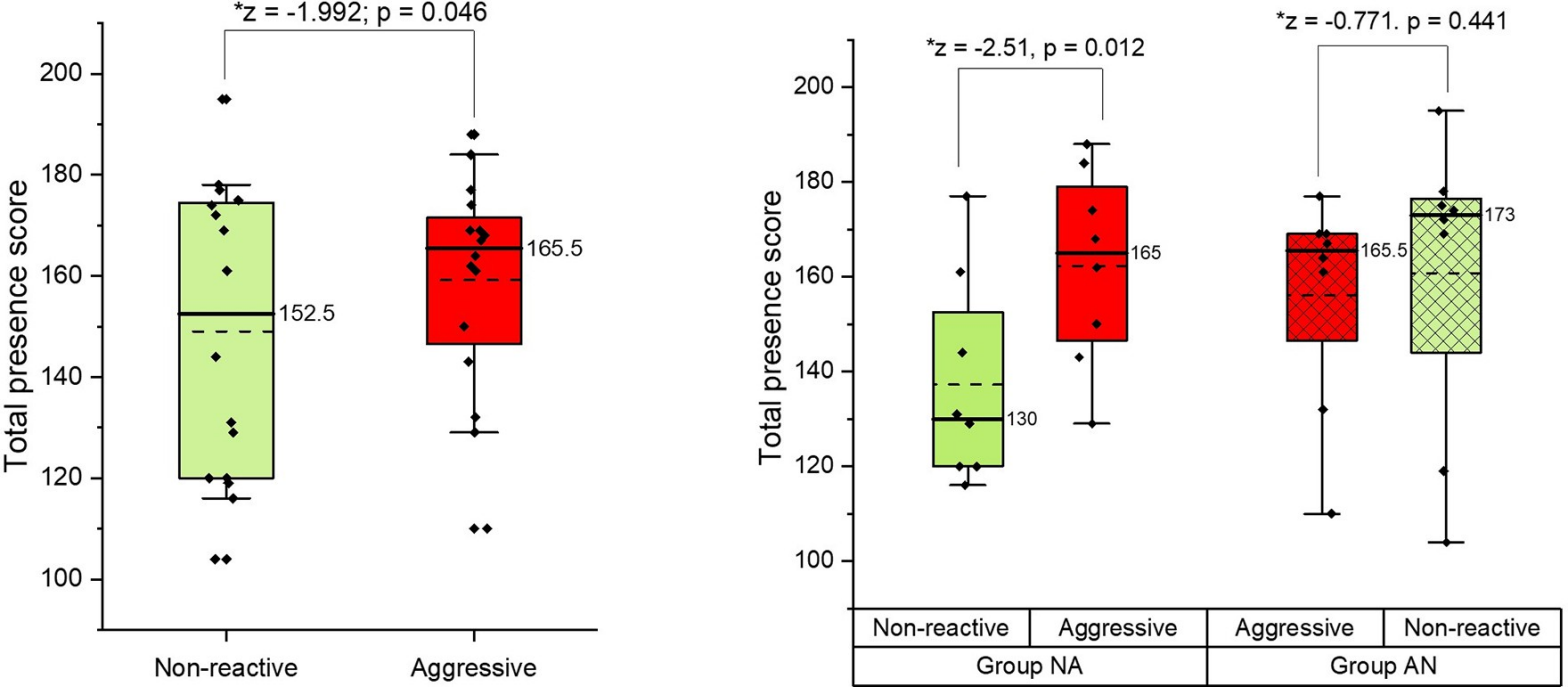
Boxplots for total presence scores and a) combined groups (NA and AN) by scenario (left) and b) comparisons between group NA and AN by scenario (right).

The mean presence score, based on Witmer *et al*. [32] presence questionnaire, was calculated (S6 Table). In the combined dataset (NA & AN), total presence scores were greater for the aggressive scenario than the non-reactive scenario (p = 0.05) (Fig 8). Within-group analysis demonstrated that this was due to a difference in total presence scores when the non-reactive scenario came first (p = 0.012) but not when the aggressive scenario came first (p = 0.441) (Fig 8).

## Discussion

The present study investigates the use of objective measures via human head and hand tracking to indicate how humans behave around a virtual dog, including one behaving aggressively towards them. This study is unique in that participants can interact with a virtual dog model via virtual reality which displays realistic behaviours based on a widely accepted theoretical model (the Canine Ladder of Aggression [27]). This is the first virtual dog model which has been reviewed by canine behavioural experts and based on the Canine Ladder of Aggression theory [27]. As recently highlighted by a systematic literature review of the current use of virtual dog models in human-animal interactions in virtual reality, Oxley *et al*. [26] found that behaviours displayed by virtual dog models varied between models, are not reviewed by canine behavioural experts and lacked accurate evidence-based representation (e.g., none of the models that displayed aggressive behaviours reported to display appeasement signals). However, it is important to note that the Canine Ladder of Aggression in practice is not as linear as theoretically displayed as dogs may show some but not all behaviours during any one incident. This was incorporated to some extent into the model based on speed of approach as levels of aggression were skipped during a fast approach (see Fig 4). In addition, this study demonstrates that the virtual dog can be used to assess human behaviour (speed and distance of approaching or avoiding) towards it. The closest distance an individual’s head and hands got to the dog appeared to be the most consistent measure between conditions, whereas gaze and total distance travelled were not. Hands of participants often came within the closest distance to the non-reactive dog model and participants stayed further away from the dog acting aggressively, however three participants were still ‘bitten’ in this test scenario.

Participants moved closer to the non-reactive dog than the aggressive dog. Participants who moved more slowly towards the aggressive dog tended also to stop earlier and at a greater distance from the dog. This could potentially indicate that either certain individuals are more cautious or can recognise relevant earlier signals more readily and therefore approach at a slower speed. Here we found no evidence of an association between demographic factors and aggression level reached, however, the sample size was small, and although there were only four males and no statistical significance, males may have tended to reach higher aggression levels. Having said this, two out of the three participants that were ‘bitten’ (level 9) were female.

The total distance individuals travelled was influenced by the order in which tasks were taken. For example, in both groups, participants travelled further in the first task compared to the second task. This could be because individuals were more cautious in the task which followed on from where the aggressive task was first (group AN). However, participants in the non-reactive task got significantly closer compared to the aggressive dog model regardless of order. In addition, both scenarios were held in the same environment for sequential five-minute periods with a short break, and participants felt they had already explored the area fully. To account for order effects future studies could consider a randomised allocation of scenarios to each participant or consider just using the aggressive scenario given that this study confirms that users react differently and approach closer to the non-reactive dog.

Regarding dog behaviour, vocalisations and gross body movement were the most common behaviours described by participants which is a similar finding to previous research [37]. Lakestani *et al*. [13] found that of the individuals (children and young adults (university students)) that correctly identified aggressive dogs through video clips, the majority (89%) did so through the identification of sounds the dog made, and likewise our participants noticed vocalisations.

It is important to note that the open-ended question responses provided different information to the close-ended questions and could highlight the perceived most important/significant or meaningful aspects of dog behaviour. When asked if they noticed specific behaviours and their meaning participants tended to describe the perceived underlying emotion or motivation (nervous, scared, threatened). Therefore, if actual specific behaviour descriptions were wanted, it would be useful to ask a separate question about the emotion(s) of the dog. In the aggressive scenario, few participants described the dog as aggressive despite three people reaching level 9 (dog lunges and bites at user). The avoidance of this term could indicate that participants attempt to justify or excuse the behaviour of the dog similar to previous research [8, 38–40]; for example, one participant stated “[the dog] had a previous negative experience” or blaming themselves one participant stated, “I was too close”. Dog behaviour (such as aggression) is often emphasised as the responsibility of the owner and not the dog [41, 42]. The attribution of the (fictional) dog having had a previous negative experience also potentially highlights the realism perceived.

The results regarding behaviour recognition are somewhat consistent with previous research. For example, just over half of the individuals in this study noticed appeasement signals such as lip licking and the head turn. The proportion of time individuals spent at aggression level 1 was low and therefore some appeasement signals could have simply not been seen. However, almost all participants agreed to the statement that they could recognise when a dog is showing aggressive or scared/fearful behaviours. Unlike Kerswell *et al*. [37], the current study used a Labrador breed dog model and thus it is unlikely that a lack of recognition of early signals was due to paedomorphic morphological characteristics such as seen in brachycephalic breeds. Four participants stated an inaccurate interpretation of lip licking and the likely meaning in this context (e.g. too hot/thirsty). There was no evidence of an association between previous/current dog ownership status and recognition of appeasement signals including lip lick and head turning in this small sample at least. Previous research agrees that both adults (including dog owners) and children, often miss early and subtle behavioural signs of aggression such as lip licking, head turning and yawning [37, 43]. Such a lack of knowledge of subtle behavioural signs is thought to be the reason for victims often stating that a dog bite was unprovoked [44, 45]. This finding highlights the need for further educational interventions which result in appropriate behavioural change and highlights the importance of recognising early behavioural signals which may be seen but misinterpreted, as well as those behaviours that may be more obvious.

Few referred to the ears of the dog, supporting previous research involving video clips of nine breeds indicating that little attention is given to this area [12], but in contrast to Demirbas *et al*. [46] who found that respondents highlighted the ears in 75% of cases when interpreting videos of behaviours in three dog breeds, Doberman, Boxer and Dalmatian. Differences in reliance on such bodily features could be due to the wide variation in ear morphology among different dog breeds, and in our Labrador, these will not have as dramatically changed in position as in some other breeds.

The tail movement was more commonly reported in the non-reactive dog than the aggressive scenario. This could have been due to the non-reactive dog continuously wagging its tail and the aggressive dog placing its tail between its legs for level 5 upwards, although shadows were also used in the model development to help identify the tail position. Tail wagging/movement has been reported elsewhere as the most reported behaviour in friendly/happy dog behaviours and emotions [12, 37]. However, tail wagging may simply indicate arousal in both positive (e.g. play) and negative (e.g. appeasement) states and the position (high, low) and type of tail wagging (stiff, relaxed, small or large movement) are likely to be more informative [47].

Given that 32% of participants had never used virtual reality, it was positive to note that after the introduction to equipment all participants could use the equipment with no additional help from the instructor. However, there were a small number of occasions identified by the instructor where participants were aware of the HMD-Laptop cable. Such incidents could briefly affect the subjective ratings of presence or result in a break in concentration and immersion (i.e. a break in presence). Other studies have reported similar issues (see [48, 49]). The presence ratings were high, indicating that both the environment and dog model were deemed to have high levels of realism and immersion. There was evidence that the aggressive dog resulted in higher levels of presence possibly due to increased interaction which was directly related to the user’s location and the dog model staring at the user.

This study provides little evidence of any cybersickness from using DAVE and thus these questionnaires are likely not needed in future research. In this study, there were only two 5-minute tasks with a 5–10-minute break to complete post-test questionnaires and VR simulator sickness has been associated with an increased length of exposure time (see review [50, 51]). Secondly, the environment was relatively stable (i.e., only the dog was moving) compared to high levels of simulator sickness noted in fast moving VR environments such as roller coasters [52]. Thirdly, the setup represented naturalistic user walking movements which has been recently noted to reduce simulator sickness in VR compared to other formats (e.g., stationary game controller movement) [51, 53]. Finally, the equipment used is of a high specification and therefore reduces the likelihood of, for example, tracking or rendering delays.

A range of further research is needed such as exploration of participant demographics and prior knowledge. For example, both the gender and personality of participants require further exploration as it is widely reported that males are at a higher risk of being bitten by dogs than females [7, 54]. Westgarth *et al*. [7] also found that in adults, being less emotionally stable was a factor associated with an increased risk of dog bites.

As the study was described ‘to investigate human behaviour around dogs’, people may have expected positive dog behaviours or human-dog interactions such as play and therefore influenced their initial response towards the dog. The perceived intention of the study could also be gathered from participants in future research. In addition, people who are familiar with VR or have an animations/virtual reality design background may be desensitised or less responsive to VR models and environments. For example, although only three people were ‘bitten’ the perception of those should be investigated further as to why they feel this occurred and who, if anyone, is to blame (e.g., participant, owner, dog, no one).

During the exploration task, the participants often stated “*I don’t know what to do*” after approximately 2–3 minutes of interaction with the dog and exploring the area. Therefore, we suggest reducing the experiment time from five to two minutes. A larger area than the current 6x2m might be useful to encourage people to explore the area, however, space availability is often a limitation with room-scale ‘real walking’ VR (when compared to controller-based, teleportation or motion-based locomotion techniques which allows larger virtual areas) [55] The application also contains a collection task, which consists of a participant being able to ‘pick up’ ten items equally placed towards the dog model, which may affect participant recognition and interaction with the dog model in both scenarios and needs testing in future research.

Given that vocalisations were one of the most recognised behaviours, further research comparing user interpretation and recognition of behaviours in scenarios with and without vocalisations would be useful. Furthermore, as previously stated a range of physical characteristics of a dog could play a role in a person’s response which could be explored such as the effect of skull shape, size, coat colour or tail movement (e.g. position, wagging frequency and different lengths). There were frequent attempts at physical interaction with the non-reactive dog, which appeared to indicate an individual attempting to stroke the virtual dog. Further development of the model could include haptics (for example tactile props (a model dog) or haptic feedback, such as controller vibration). Verbal communication directed towards the dog model was also noted in the current study, as reported in previous VR and AR related research involving both AR and VR dog models. For example, Norouzi *et al*. [25] found that participants called the dog’s name or used terms to get the attention of the dog. Future studies should record verbal comments and physical and emotional reactions during the task (e.g. in one case a person jumped and screamed when quickly approaching the aggressive dog which lunged), including at what time and during what scenario they occurred.

This research highlights the potential application into various aspects of injury prevention education. For example, Tulloch *et al*. [4] recently reported that adult hospital admissions in England due to dog bites have tripled between 1998 and 2018. Therefore, the model reported in the present study could be incorporated into dog bite prevention education to teach and assess adults about the relevant canine behaviour signals which may be displayed prior to a bite such as those displayed in the Canine Ladder of Aggression. Furthermore, the non-reactive dog model can also be incorporated into dog phobia treatments as a form of gradual exposure [26]. The current virtual reality navigation in the present study (room scale movement) differs from previous phobia treatments (mouse/joystick) and is therefore likely to be more realistic for the user [26]. Furthermore, those individuals who are in occupations that may be at higher risk of dog bite injuries, such as postal and kennel workers, veterinary students/staff, could also benefit from education about dog behaviour in a real time and risk-free environment in virtual reality. Therefore, the use of the DAVE model has potential to be used as both an assessment and prevention/intervention tool for various audiences.

This study was not without limitations. The sample consisted of only university students, however, this included both undergraduate and postgraduate from a range of backgrounds and areas of study. Further studies could select a range of adults and children to take part to explore the effects of different ages. Most participants were female, a gender bias often seen in studies relating to human-animal interactions [56]. Interestingly, in contrast, VR studies often report a bias in male participants [57]. The majority (10/16) had currently or previously owned dogs which may indicate some form of self-selection bias. Furthermore, participants were not asked if they had previously taken part in dog safety training which may have resulted in differing behaviour and approach distances towards both aggressive and non-reactive dogs. Three individuals had been previously bitten by a dog; however, no definition of a bite was provided, or the seriousness and context of the incident were discussed. Future research needs to include a definition of what is considered a dog bite (e.g., ‘a bruise or puncture to the skin’ [58]). Having said this, all participants were screened to ensure they were not fearful/scared, anxious or phobic of dogs.

It is important to note that only a single coordinate on the dog model was placed on the dog’s nose. Therefore, more points are needed to ascertain how close individual hands get to other areas of the dog (e.g. the back of the head). The dog model was limited to the reaction to a person based on their speed of approach and distance of the participant. In a real-world scenario, a dog may perceive threats by a participant that were not included such as eye contact, facial expression, posture and specific behaviours [59], which could be development ideas for the future as technology advances. Having said this, the research presented in this study would be unlikely to have been conducted in a real world setting due to the welfare concerns for both human and dogs. Future research could incorporate the use of a dog displaying positive interactions such as the dog approaching the user, rather than the dog being non-reactive/neutral. In addition, further areas would be useful to research including different scenarios (e.g., the dog sleeping on a bed), environment (e.g., outdoor park), and tasks (e.g. move the dog).

The recognition of yawning was not included due to trying to minimise the length of the questionnaire. However, it was mentioned by several participants either by observing the behaviour or hearing the vocalisation. Some behaviours which may be evident in a real dog were not present due to development limitations, such as piloerection which has previously been reported in aggressive behaviours, but often are not identified by participants [12].

## Conclusion

This study tested a range of objective and subjective measures which were modulated by the dog models non-reactive and aggressive conditions. Participants moved significantly closer to the non-reactive dog model compared to the aggressive dog model, indicating they perceived it as less of a threat to them, as supported by their reported interpretations. Participants most often focused on body movements when describing behaviours and often stated emotional or motivational justifications for behaviours seen, and also noticed vocalisations. Participants appeared to perceive the dog as realistic and act in a manner similar to what might be expected during an interaction with a live dog, and simulator sickness was not an issue. This study highlights the potential future use of such a model for the purposes of interventions (e.g. dog phobia treatment), evaluating interventions (e.g. dog safety education) and research (e.g. human behaviour in the presence of dogs).

## Supporting information

S1 Fig The highest-level participants reached, and the time taken to reach the highest level. (PNG)

S2 Fig A boxplot comparison displaying the median time participants in each group (NA/AN) spent in each level of aggression (0–9) over the five-minute aggression task. (PNG)

S1 Table Survey questions about dog behaviour recognition and interpretation. (DOCX)

S2 Table Participants responses to six statements relating to their feelings in the presence of dogs and perceived ability to recognise dog behaviours. (DOCX)

S3 Table Categorised open-ended questions when asked to describe overall behaviour (“Describe what you think the behaviour(s) indicated about the dog“) (multiple descriptors allowed per respondent). (DOCX)

S4 Table Perceived meaning of specific behaviours (lip lick, paw raise, head turn, backing away and showing teeth) regardless of whether a participant saw the behaviour during the scenario or not (n = 16). (Multiple responses were allowed per person).
(DOCX)

S5 Table A Friedmann test found that there was no significant difference between total mean weighted scores (n = 16) by either scenario (pre-test-non-reactive-aggressive) (p = 0.387) or by order of tasks (pre-test-1^st^ task-2^nd^ task) (p = 0.911). Furthermore, there was no significant difference in total scores within group NA (pre-test-aggressive-non-reactive) (p = 0.905) or within group AN (p = 0.61). Weighted SSQ scores including nausea, oculomotor, disorientation subscales and total scores.
(DOCX)

S6 Table Subjective mean scores and standard deviation from the 29-item presence questionnaire split by the four-factor model (Witmer *et al*., 2005) with internal consistency (Cronbach’s alpha (C *α*)). (DOCX)

## Abbreviations

VR: Virtual Reality
AR: Augmented Reality
DAVE: Dog Assisted Virtual Environment
AN: Aggressive Non-reactive group
NA: Non-reactive Aggressive group
HMD: Head Mounted Display
