# Supplementary figures and images for: A pilot study investigating human behaviour towards DAVE (Dog Assisted Virtual Environment) and interpretation of non-reactive and aggressive behaviours during a virtual reality exploration task
Source: PLoS One. 2022 Sep 28;17(9):e0274329. doi: 10.1371/journal.pone.0274329 (PMC9518854; doi:10.1371/journal.pone.0274329)

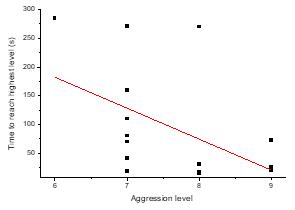

Supplement: S1 Fig — (PNG) [file pone.0274329.s001.png]

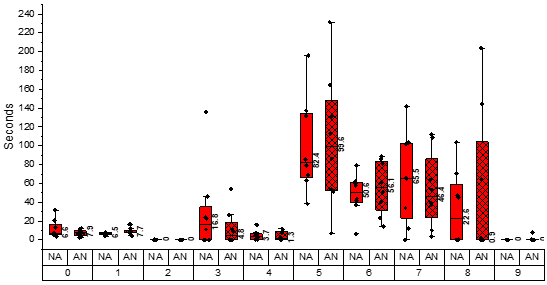

Supplement: S2 Fig — (PNG) [file pone.0274329.s002.png]
