# Supplementary material for: A pilot study investigating human behaviour towards DAVE (Dog Assisted Virtual Environment) and interpretation of non-reactive and aggressive behaviours during a virtual reality exploration task
Source: PLoS One. 2022 Sep 28;17(9):e0274329. doi: 10.1371/journal.pone.0274329 (PMC9518854; doi:10.1371/journal.pone.0274329)
Supplement: S1 Table — (DOCX) [file pone.0274329.s003.docx]

**S1 Table**

| **Questions** | | **Answers** |
| --- | --- | --- |
| Did you notice anything about the behaviour of the dog? | | *Open ended* |
| What do you think the behaviour indicated about the dog? | | *Open ended* |
| Did you see the dog [Behaviour]? | | *Yes, No*  *[For each behaviour with an image of the behaviour being displayed in the relevant scenario]* |
| **Aggressive:**  ‘Lip lick’, ‘raise its paw’, ‘lying down’, ‘turn its head away’, ‘backing away’, ‘showing its teeth’, ‘standing’ | **Non-reactive:**  ‘lying down’  ‘standing’ |  |
| What do you think this behaviour meant? | | *Open ended [for each behaviour]* |
| Did you notice any dog related sounds/vocalisations? | | *Yes/No [Aggression only]* |
| If Yes, please describe what you heard and what you thought these sounds/vocalisations meant? | | *Open ended [Aggression only]* |
