# Supplementary material for: A pilot study investigating human behaviour towards DAVE (Dog Assisted Virtual Environment) and interpretation of non-reactive and aggressive behaviours during a virtual reality exploration task
Source: PLoS One. 2022 Sep 28;17(9):e0274329. doi: 10.1371/journal.pone.0274329 (PMC9518854; doi:10.1371/journal.pone.0274329)
Supplement: S2 Table — (DOCX) [file pone.0274329.s004.docx]

**S2 Table**

| **Statement** | **Strongly  Disagree  n (%)** | **Disagree  n (%)** | **Neither  n (%)** | **Agree  n (%)** | **Strongly  Agree n (%)** | **Total n (%)** |
| --- | --- | --- | --- | --- | --- | --- |
| 1. *“I am cautious in presence of most* *dogs*” | 7 (43.75) | 6 (37.50) | 1 (6.25) | 2 (12.50) | 0 (0) | 16 (100) |
| 2. “*I enjoy the presence of most dogs”* | 0 (0) | 0 (0) | 2 (12.50) | 2 (12.50) | 12 (75.00) | 16 (100) |
| 3. “*I feel relaxed in the presence of* *most dogs*” | 0 (0) | 0 (0) | 3 (18.75) | 5 (31.25) | 8 (50.00) | 16 (100) |
| 4. “*I can recognise when a dog is showing aggressive* *behaviours*” | 0 (0) | 1 (6.25) | 0 (0) | 11 (68.75) | 4 (25.00) | 16 (100) |
| 5. *“I can recognise when a dog is showing scared/fearful behaviours”* | 0 (0) | 2 (12.50) | 1 (6.25) | 9 (56.25) | 4 (25.00) | 16 (100) |
| 6. “*I can recognise when a dog is showing relaxed behaviours*” | 0 (0) | 0 (0) | 1 (6.25) | 9 (56.25) | 6 (37.50) | 16 (100) |
