# Supplementary material for: A pilot study investigating human behaviour towards DAVE (Dog Assisted Virtual Environment) and interpretation of non-reactive and aggressive behaviours during a virtual reality exploration task
Source: PLoS One. 2022 Sep 28;17(9):e0274329. doi: 10.1371/journal.pone.0274329 (PMC9518854; doi:10.1371/journal.pone.0274329)
Supplement: S3 Table — (DOCX) [file pone.0274329.s005.docx]

**S3 Table**

| **Non-reactive Scenario** | **n** |  | **Aggressive Scenario** | **n** |
| --- | --- | --- | --- | --- |
| Relaxed, comfortable, calm, happy, nice | 8 |  | Nervous, anxious, stressed, scared/unsure around people, timid, afraid | 13 |
| Wanted attention, loving, waiting for someone | 4 |  | Threatened | 3 |
| Anxious, nervous | 2 |  | Negative previous experience/treatment | 3 |
| Yawned and whined | 1 |  | Defensive/ready to defend | 3 |
| Unhappy | 1 |  | Unfriendly/ Not in a good mood | 2 |
| Wagging tail and breathing | 1 |  | Confused | 1 |
| Uncomfortable | 1 |  | Aggressive | 1 |
|  |  |  | Not confident | 1 |
|  |  |  | Lost | 1 |
