# Supplementary material for: A pilot study investigating human behaviour towards DAVE (Dog Assisted Virtual Environment) and interpretation of non-reactive and aggressive behaviours during a virtual reality exploration task
Source: PLoS One. 2022 Sep 28;17(9):e0274329. doi: 10.1371/journal.pone.0274329 (PMC9518854; doi:10.1371/journal.pone.0274329)
Supplement: S4 Table — (Multiple responses were allowed per person). (DOCX) [file pone.0274329.s006.docx]

**S4 Table.**

| **Behaviours** | **n** |
| --- | --- |
| **Lip Lick** |  |
| Hungry or thirsty / Too hot | 4 |
| Nervous / Unsure / Anxious | 3 |
| Warning to back off / Ready to defend itself | 2 |
| Aggressive | 1 |
| Comfortable | 1 |
| Did not answer the question | 5 |
|  |  |
| **Paw Raise** |  |
| Anxious / Worried / Scared / Wary / Unsure | 6 |
| Actual or preparation for movement towards/away from the user (e.g. Preparing to move / lunge / run away / 'come at you') | 5 |
| Submission | 2 |
| Attention (e.g. "He wanted something") | 1 |
| Beginning to feel at ease | 1 |
| Enticing (e.g. "At first it looked like it was inviting...") | 1 |
| Hurt paw | 1 |
| Defensive | 1 |
| Saying hello with their paw | 1 |
| Waiting on user response to see if they would get closer | 1 |
|  |  |
| **Head Turn** |  |
| Looking around the environment for threats/distracted/disengaged | 5 |
| Lack of eye contact (e.g. Does not like eye contact / No eye contact) | 3 |
| Did not want to interact | 2 |
| Threatened (e.g., Perceived user as a threat) | 2 |
| Feeling safe | 1 |
| Following a moving object (e.g., user moving arms) | 1 |
| Submission | 1 |
| Nervous / Unsure | 1 |
| Did not answer the question | 4 |
|  |  |
| **Backing away** |  |
| Nervous / Anxious / Scared / Frightened / Wary / Unsure / Distressed | 8 |
| Dog perceived user as a threat / distance increasing behaviour (e.g. dog moved back as user moved forward / Removed itself from the situation) | 9 |
| Defensive | 2 |
| Snarled | 1 |
| Did not answer the question | 1 |
|  |  |
| **Showing its teeth** |  |
| Trying to get user to move away (e.g. Warning to back away / I was too close / Trying to make me leave) | 8 |
| Aggressive | 5 |
| Frustrated / Angry / Scared / Upset / Not happy | 4 |
| Trying to intimidate / scare me | 2 |
| Warning behaviour / sign | 2 |
| Defensive / defend itself | 2 |
| Territorial | 1 |
