# Supplementary material for: A pilot study investigating human behaviour towards DAVE (Dog Assisted Virtual Environment) and interpretation of non-reactive and aggressive behaviours during a virtual reality exploration task
Source: PLoS One. 2022 Sep 28;17(9):e0274329. doi: 10.1371/journal.pone.0274329 (PMC9518854; doi:10.1371/journal.pone.0274329)
Supplement: S5 Table — Furthermore, there was no significant difference in total scores within group NA (pre-test-aggressive-non-reactive) (p = 0.905) or within group AN (p = 0.61). Weighted SSQ scores including nausea, oculomotor, disorientation subscales and total scores. (DOCX) [file pone.0274329.s007.docx]

**S5 Table.**

|  | **Pre-Test SSQ score** | | | **Non-reactive SSQ score** | | | **Aggressive SSQ Score** | | |
| --- | --- | --- | --- | --- | --- | --- | --- | --- | --- |
| **Subscale** | **Mean** | **S.E.** | **S.D.** | **Mean** | **S.E.** | **S.D.** | **Mean** | **S.E.** | **S.D.** |
| **Nausea** | 2.98 | 1.14 | 4.57 | 3.58 | 1.48 | 5.91 | 5.37 | 2.30 | 9.20 |
| **Oculomotor** | 5.69 | 1.62 | 6.49 | 5.69 | 1.90 | 7.58 | 8.53 | 3.01 | 12.02 |
| **Disorientation** | 0.87 | 0.87 | 3.48 | 4.35 | 2.10 | 8.38 | 3.48 | 2.01 | 8.04 |
| **Total*** | 4.21 | 1.18 | 4.71 | 5.38 | 1.74 | 6.96 | 7.25 | 2.59 | 10.35 |
| **Task order** | **Pre-Test** | | | **1^st^ Task** | | | **2^nd^ Task** | | |
| **Nausea** | 2.98 | 1.14 | 4.87 | 3.58 | 1.48 | 5.91 | 5.37 | 2.30 | 9.20 |
| **Oculomotor** | 5.69 | 1.62 | 6.49 | 7.11 | 2.01 | 8.05 | 7.58 | 2.68 | 10.27 |
| **Disorientation** | 0.87 | 0.87 | 3.48 | 3.48 | 2.01 | 8.04 | 4.35 | 2.10 | 8.38 |
| **Total*** | 4.21 | 1.18 | 4.71 | 5.84 | 1.74 | 6.96 | 7.01 | 2.18 | 8.73 |

**Total score is calculated by the addition of nausea, oculomotor and disorientation raw (unweighted) scores and multiplied by 3.74 (Kennedy et al., 1993).*
