# Supplementary material for: A pilot study investigating human behaviour towards DAVE (Dog Assisted Virtual Environment) and interpretation of non-reactive and aggressive behaviours during a virtual reality exploration task
Source: PLoS One. 2022 Sep 28;17(9):e0274329. doi: 10.1371/journal.pone.0274329 (PMC9518854; doi:10.1371/journal.pone.0274329)
Supplement: S6 Table — (DOCX) [file pone.0274329.s008.docx]

**Table 6**

|  |  | | **Aggressive (n=16)** | | | |  | | **Non-reactive (n=16)** | | | |
| --- | --- | --- | --- | --- | --- | --- | --- | --- | --- | --- | --- | --- |
| **Factor** | **Item** | **Mean** | | **SD** | **Factor mean. (SD)** | **C *α*** | **Item** | **Mean** | | **SD** | **Factor**  **mean. (SD)** | **C *α*** |
| **Involvement** | 1 | 4.69 | | 1.537 | 5.20 (0.977) | 0.857 | 1 | 3.56 | | 2.220 | 4.76 (1.045) | 0.931 |
|  | 2 | 5.56 | | 0.892 |  |  | 2 | 3.88 | | 2.125 |  |  |
|  | 3 | 4.75 | | 1.528 |  |  | 3 | 4.44 | | 2.159 |  |  |
|  | 4 | 5.44 | | 0.964 |  |  | 4 | 4.81 | | 1.682 |  |  |
|  | 6 | 5.88 | | 1.408 |  |  | 6 | 5.75 | | 1.653 |  |  |
|  | 7 | 5.63 | | 1.147 |  |  | 7 | 5.38 | | 1.258 |  |  |
|  | 8 | 5.44 | | 1.413 |  |  | 8 | 5.00 | | 1.366 |  |  |
|  | 10 | 6.25 | | 0.775 |  |  | 10 | 6.06 | | 0.998 |  |  |
|  | 14 | 6.06 | | 1.124 |  |  | 14 | 6.00 | | 1.095 |  |  |
|  | 17 | 2.94 | | 1.611 |  |  | 17 | 2.63 | | 1.628 |  |  |
|  | 18 | 5.88 | | 1.025 |  |  | 18 | 5.31 | | 1.537 |  |  |
|  | 26 | 3.88 | | 1.996 |  |  | 26 | 4.31 | | 2.358 |  |  |
| **Sensory Fidelity** | 5 | 5.50 | | 0.894 | 5.02  (1.252) | 0.784 | 5 | 4.69 | | 1.740 | 4.96  (0.852) | 0.772 |
|  | 11 | 6.44 | | 0.727 |  |  | 11 | 5.94 | | 1.063 |  |  |
|  | 12 | 5.94 | | 0.854 |  |  | 12 | 5.44 | | 1.153 |  |  |
|  | 13 | 2.94 | | 1.879 |  |  | 13 | 3.44 | | 2.032 |  |  |
|  | 15 | 4.38 | | 1.408 |  |  | 15 | 5.13 | | 1.408 |  |  |
|  | 16 | 4.94 | | 1.340 |  |  | 16 | 5.13 | | 1.408 |  |  |
| **Adaption/ Immersion** | 9 | 5.31 | | 1.621 | 5.99  (0.378) | 0.822 | 9 | 3.63 | | 1.996 | 5.57  (0.859) | 0.859 |
|  | 20 | 6.63 | | 0.619 |  |  | 20 | 6.63 | | 0.806 |  |  |
|  | 21 | 5.69 | | 1.448 |  |  | 21 | 5.75 | | 1.342 |  |  |
|  | 24 | 5.94 | | 1.181 |  |  | 24 | 5.56 | | 1.153 |  |  |
|  | 25 | 6.06 | | 0.772 |  |  | 25 | 5.56 | | 1.031 |  |  |
|  | 27 | 6.06 | | 1.063 |  |  | 27 | 5.63 | | 1.360 |  |  |
|  | 28 | 6.13 | | 1.408 |  |  | 28 | 6.00 | | 1.265 |  |  |
|  | 29 | 6.06 | | 0.854 |  |  | 29 | 5.81 | | 0.981 |  |  |
| **Interface quality** | 19 | 6.13 | | 0.885 | 6.27 (0.129) | 0.603 | 19 | 6.13 | | 1.088 | 6.15  (0.035) | 0.387 |
|  | 22 | 6.38 | | 1.025 |  |  | 22 | 6.13 | | 0.957 |  |  |
|  | 23 | 6.31 | | 0.946 |  |  | 23 | 6.19 | | 0.981 |  |  |
